# Pelvic Lymph Node Dissection During Cystectomy for Patients With Bladder Carcinoma With Variant Histology: Does Histologic Type Matter?

**DOI:** 10.3389/fonc.2020.545921

**Published:** 2020-10-19

**Authors:** Lijuan Guo, Lianghao Zhang, Jiange Wang, Xuepei Zhang, Zhaowei Zhu

**Affiliations:** ^1^Department of Disease Prevention and Control, The First Affiliated Hospital of Zhengzhou University, Zhengzhou, China; ^2^Department of Urology, The First Affiliated Hospital of Zhengzhou University, Zhengzhou, China

**Keywords:** bladder neoplasms, cystectomy, histologic types, pelvic lymph node dissection, survival

## Abstract

**Purpose:**

Adding pelvic lymph node dissection (PLND) to cystectomy offers significant survival benefit. However, it remains unclear whether this benefit persists in all histologic types. The aim of the study was to examine the impact of PLND on overall survival (OS) after cystectomy in bladder carcinoma patients with histological variants.

**Methods:**

Within the Surveillance, Epidemiology and End Results database, we identified 16,880 bladder carcinoma patients receiving cystectomy between 2004 and 2015. Patients were stratified according to the following histologic types: transitional cell carcinoma, squamous cell carcinoma, adenocarcinoma, small cell carcinoma, neuroendocrine carcinoma, signet ring cell carcinoma, pseudosarcomatous carcinoma, and other histology. Cox regression models were used to evaluate the effect of PLND on OS stratified by histologic type.

**Results:**

Histologic types were significantly associated with the presence of lymph node metastasis in patients with bladder carcinoma (*P* < 0.001). In multivariable Cox regression analyses, PLND compared with non-PLND was associated with OS benefit in patients with transitional cell carcinoma (hazard ratio [HR], 0.595; 95% confidence interval [95% CI], 0.557–0.634 [*P* < 0.001]), squamous cell carcinoma (HR, 0.646; 95% CI, 0.494–0.846 [*P* = 0.002]), and signet ring cell carcinoma (HR, 0.233; 95% CI, 0.107–0.504 [*P* < 0.001]), whereas no significant differences in OS were observed in other histological subsets.

**Discussion:**

Our analyses revealed a significant OS benefit from PLND in patients with transitional cell carcinoma, squamous cell carcinoma, and signet ring cell carcinoma. However, a survival benefit of PLND in patients with other histologic types was not demonstrated.

## Introduction

Bladder carcinoma is one of the most common cancers worldwide, with an estimated 81,400 new patients diagnosed in the United States in 2020 (1). Radical cystectomy is the main surgical modality for high-risk non-muscle-invasive and muscle-invasive bladder cancer (2, 3). Noteworthy, almost 25% of bladder cancer patients receiving cystectomy have lymph node invasion (4). Thus, increasing evidence suggests that adding pelvic lymph node dissection (PLND) to cystectomy provides considerable benefits, which are emphasized by providing accurate nodal staging and reducing disease burden (5, 6).

Although urothelial (transitional cell) carcinoma is the most common pathological type, non-urothelial variants are observed in up to a quarter of patients and represent a rare and challenging group (7–9). Notably, non-urothelial histologic types have been associated with biologically aggressive disease and unfavorable survival outcomes compared with urothelial carcinoma of the bladder (7–9). However, current research supporting PLND in the treatment of bladder carcinoma is largely based on studies limited to urothelial carcinoma (10). Evidence concerning PLND in bladder cancer patients with non-urothelial histological variants is limited and inconsistent.

To the best of our knowledge, the impact of variant histologic types on the presence of lymph node invasion and oncological outcomes following cystectomy are currently undetermined. Based on these considerations, we sought to assess the relation between histologic type and lymph node status after cystectomy using the Surveillance, Epidemiology, and End Results (SEER) database. According to histologic type, we also examined the effect of PLND on overall survival (OS) between patients receiving cystectomy and PLND versus those receiving cystectomy alone.

## Materials and Methods

### Data Source

The SEER database uses data from 18 population-based state registries representing roughly 30% of the United States population. The comprehensive database adopts the International Classification of Disease for Oncology, third edition (ICD-O-3) for histology coding. Bladder cancer cases diagnosed between 2004 and 2015 were ascertained from the SEER database and their demographic and clinical characteristics were retrieved using SEER^∗^Stat version 8.3.6 software.

### Study Population and Treatment Groups

**FIGURE 1:**
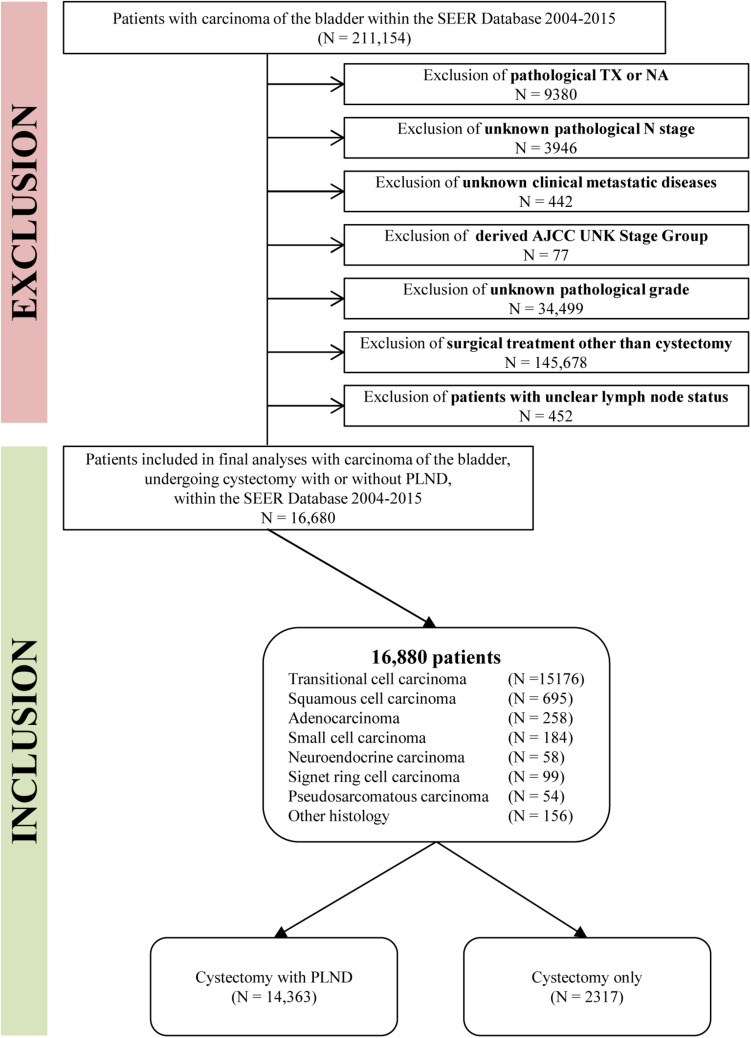
Flowchart describing the selection of bladder cancer patients who underwent cystectomy with or without pelvic lymph node dissection (PLND) within the SEER from 2004 to 2015.

In total, 211,154 patients were diagnosed with bladder carcinoma between 2004 and 2015 within the SEER database. According to the American Joint Committee on Cancer staging system, we only considered bladder cancer cases with known tumor stage and grade. Another inclusion criterion was the acceptance of cystectomy as the main surgical treatment. Patients were excluded if it was unknown whether PLND was performed. Patients with Nx disease were also excluded due to unclear lymph node status. Detailed exclusion criteria are shown in Figure 1.

Additionally, we used the “Regional nodes examined” variable to distinguish between cases who underwent PLND and those who did not. Patients were stratified according to the following histologic types: transitional cell carcinoma, squamous cell carcinoma, adenocarcinoma, small cell carcinoma, neuroendocrine carcinoma, signet ring cell carcinoma, pseudosarcomatous carcinoma, and other histology.

### Covariates

Patient demographics and clinical characteristics are shown in Table 1. Patient covariates included demographics (age, sex, race), tumor stage and grade. According to the TNM classification system, the tumor stage was categorized as T0, Ta, Tis, T1, T2, T3, or T4, and the lymph node status was classified as N0 or N+ (≥N1). The pathological grade was subdivided into well differentiated (Grade I), moderately differentiated (Grade II), poorly differentiated (Grade III), or undifferentiated, anaplastic (Grade IV).

**TABLE 1:** Demographic and clinical characteristics of 16,880 patients undergoing cystectomy stratified by PLND versus no PLND within the SEER between 2004 and 2015.

	No. of Patients (%)^*a*^			
Characteristic	Overall, *N* = 16,880	PLND, *N* = 14,363	No PLND, *N* = 2317	*P*
Age: Mean ± SD, y	67.76 ± 10.58	67.55 ± 10.50	69.09 ± 10.98	<0.001
**Sex**				
Men	12481 (74.8)	10764 (74.9)	1717 (74.1)	0.395
Women	4199 (25.2)	3599 (25.1)	600 (25.9)	
Race				
White	14794 (88.7)	12718 (88.5)	2076 (89.6)	0.048
Black	1042 (6.2)	891 (6.2)	151 (6.5)	
Other	816 (4.9)	729 (5.1)	87 (3.8)	
Unknown	28 (0.2)	25 (0.2)	3 (0.1)	
**Histologic type**				
Transitional cell carcinoma	15176 (91.0)	13078 (91.1)	2098 (90.5)	0.007
Squamous cell carcinoma	695 (4.2)	598 (4.2)	97 (4.2)	
Adenocarcinoma	258 (1.5)	215 (1.5)	43 (1.9)	
Small cell carcinoma	184 (1.1)	169 (1.2)	15 (0.6)	
Neuroendocrine carcinoma	58 (0.3)	53 (0.4)	5 (0.2)	
Signet ring cell carcinoma	99 (0.6)	85 (0.6)	14 (0.6)	
Pseudosarcomatous carcinoma	54 (0.3)	45 (0.3)	9 (0.4)	
Other histology	156 (0.9)	120 (0.8)	36 (1.6)	
**Grade**				
I	183 (1.1)	120 (0.8)	63 (2.7)	<0.001
II	889 (5.3)	681 (4.7)	208 (9.0)	
III	5083 (30.5)	4300 (29.9)	783 (33.8)	
IV	10525 (63.1)	9262 (64.5)	1263 (54.5)	
**Derived AJCC T, 6th ed**				
≤T1	2247 (13.5)	1630 (11.3)	617 (26.6)	<0.001
T2	6282 (37.7)	5319 (37.0)	963 (41.6)	
T3	5204 (31.2)	4829 (33.6)	375 (16.2)	
T4	2947 (17.7)	2585 (18.0)	362 (15.6)	
**Derived AJCC N, 6th ed**				
N0	12550 (75.2)	10285 (71.6)	2265 (97.8)	<0.001
N1	2063 (12.4)	2031 (14.1)	32 (1.4)	
N2	1990 (11.9)	1970 (13.7)	20 (0.9)	
N3	77 (0.5)	77 (0.5)	0 (0.0)	

*AJCC, American Joint Committee on Cancer; SD, standard deviation of the mean; PLND, pelvic lymph node dissection. ^*a*^ Percentages may not add up to 100% because they were not reported completely.*

### Outcome

Our outcome of interest was OS. Survival months were calculated from the date of cystectomy to the date of last contact or death. We compared OS between cases who underwent cystectomy and PLND versus those who received cystectomy without PLND, stratified by histologic type.

### Statistical Analyses

Means and standard deviations were reported for continuous variables, and frequencies and percentages were reported for categorical variables. We used the Student *t* test and chi-square test to assess differences between treatment groups.

Kaplan-Meier curves were constructed to compare OS between patients who received cystectomy and PLND versus those who received cystectomy only. The log-rank test was used to test differences in OS between treatment groups. Cox regression analyses were carried out to compute hazard ratio (HR) for the impact of PLND on OS for different histologic types of bladder carcinoma. Multivariable Cox regression analyses were adjusted for demographics (age, sex, race), stage group, tumor stage and grade.

All statistical analyses in the current study were performed using SPSS statistical software (Version 20.0; SPSS Inc, Chicago, IL, United States). All statistical tests were two-sided, and *P* values < 0.05 were considered statistically significant.

## Results

### Study Cohort and Baseline Characteristics

Overall, 16,880 patients met our inclusion criteria, and most had transitional cell carcinoma (91.0%; 15,176 of 16,880 patients). Within the entire cohort, 86.1% (14363 of 16,880 patients) received cystectomy and PLND, compared with 13.9% (2317 of 16,880 patients) who underwent cystectomy without PLND.

When analyzing patients according to whether PLND was performed, patients in the PLND group were younger (67.55 vs. 69.09 years; *P* < 0.001), were more often correlated with advanced stage and grade (*P* < 0.001), and had more lymph node metastasis (28.3% vs. 2.3%; *P* < 0.001). A detailed comparison of patients in the PNLD and non-PLND group is provided in Table 1.

Comparing patients with different histologic types, lymph node metastasis at the time of cystectomy were 28.0% (3656 of 13078 patients) for transitional cell carcinoma, 27.3% (163 of 598 patients) for squamous cell carcinoma, 33.0% (71 of 215 patients) for adenocarcinoma, 35.5% (60 of 169 patients) for small cell carcinoma, 34.0% (18 of 53 patients) for neuroendocrine carcinoma, 58.8% (50 of 85 patients) for signet ring cell carcinoma, and 17.8% (8 of 45 patients) for pseudosarcomatous carcinoma, respectively (Table 2). Variant histologic types were significantly associated with the presence of lymph node metastasis in patients with bladder cancer (*P* < 0.001).

**TABLE 2:** Lymph-Node-Positive disease at cystectomy and PLND in each histologic type within the SEER from 2004 to 2015.

	No. of Patients (%)^*a*^			
Histologic type	Overall, *N* = 14363	LN negative, *N* = 10302	LN positive, *N* = 4061	*P*
Transitional cell carcinoma	13078	9422 (72.0)	3656 (28.0)	<0.001
Squamous cell carcinoma	598	435 (72.7)	163 (27.3)	
Adenocarcinoma	215	144 (67.0)	71 (33.0)	
Small cell carcinoma	169	109 (64.5)	60 (35.5)	
Neuroendocrine carcinoma	53	35 (66.0)	18 (34.0)	
Signet ring cell carcinoma	85	35 (41.2)	50 (58.8)	
Pseudosarcomatous carcinoma	45	37 (82.2)	8 (17.8)	
Other histology	120	85 (70.8)	35 (29.2)	

*LN, lymph node; PLDN, pelvic lymph node dissection.*

### Survival Analyses

**FIGURE 2:**
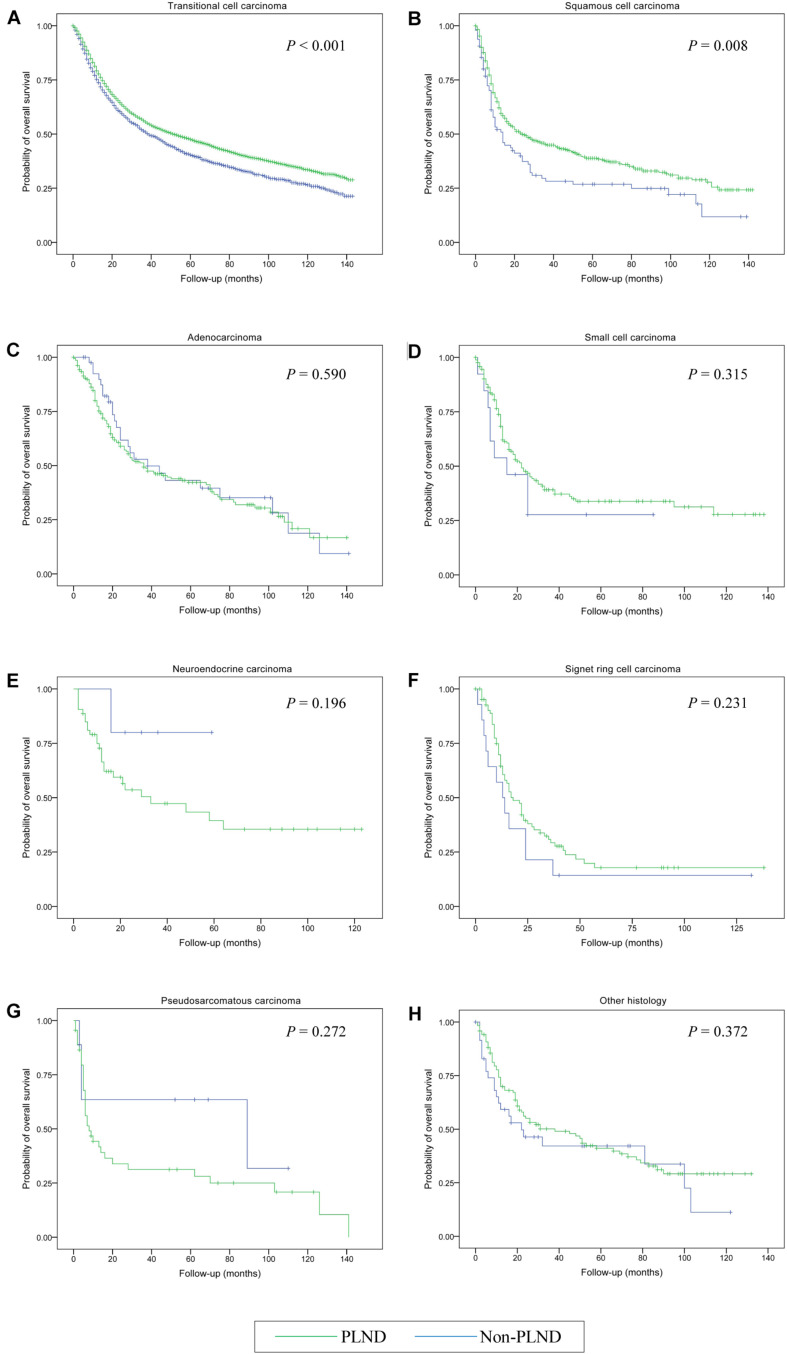
Kaplan-Meier analysis of overall survival in patients who received cystectomy and pelvic lymph node dissection (PLND) versus cystectomy only for the treatment of bladder cancer with different histologic types. **(A)** Transitional cell carcinoma; **(B)** Squamous cell carcinoma; **(C)** Adenocarcinoma; **(D)** Small cell carcinoma; **(E)** Neuroendocrine carcinoma; **(F)** Signet ring cell carcinoma; **(G)** Pseudosarcomatous carcinoma; **(H)** Other histology.

The median OS for the entire cohort was 47.00 months (95% CI, 44.74–49.26 months). Cystectomy with PLND was associated with better OS compared with non-PLND cohorts (49.00 vs. 37.00 months; *P* < 0.001). Table 3 provides details of the median OS for each histologic type stratified by treatment group. In univariable Cox Regression analyses, a difference in OS for PLND versus non-PLND cohorts was only observed for transitional cell carcinoma (52.00 vs. 39.00 months; *P* < 0.001) and squamous cell carcinoma (24.00 vs. 14.00 months; *P* = 0.008) whereas there was no statistically significant difference in OS for other histologic types (Figure 2).

**TABLE 3:** Median overall survival in patients with bladder carcinoma and variant histology within the SEER from 2004 to 2015.

	Median OS (95% CI), mo ^*a*^	
Histologic type	PLND	No PLND
Transitional cell carcinoma	52.00 (49.04–54.96)	39.00 (34.79–43.21)
Squamous cell carcinoma	24.00 (16.04–31.96)	14.00 (9.19–18.81)
Adenocarcinoma	36.00 (22.32–49.68)	38.00 (14.46–61.54)
Small cell carcinoma	22.00 (15.35–28.65)	15.00 (0.59–29.41)
Neuroendocrine carcinoma	33.00 (1.61–64.39)	/
Signet ring cell carcinoma	18.00 (12.62–23.38)	13.00 (5.67–20.33)
Pseudosarcomatous carcinoma	8.00 (2.94–13.06)	89.00 (0.00–214.89)
Other histology	38.00 (16.16–59.84)	22.00 (2.49–41.51)
Overall	49.00 (46.27–51.73)	37.00 (33.13–40.87)

*95% CI, 95% confidence interval; OS, overall survival; PLND, pelvic lymph node dissection.*

Adjusted multivariable Cox regression analyses demonstrated a significant OS benefit for cystectomy and PLND in patients with transitional cell carcinoma (HR, 0.595; 95% CI, 0.557–0.634 [*P* < 0.001]), squamous cell carcinoma (HR, 0.646; 95% CI, 0.494–0.846 [*P* = 0.002]), and signet ring cell carcinoma (HR, 0.233; 95% CI, 0.107–0.504 [*P* < 0.001]) compared with patients undergoing cystectomy only. Conversely, there were no significant differences in OS noted between the treatment groups in any other histological subsets (all *P* > 0.05) (Table 4).

**TABLE 4:** Overall mortality rate, univariable and multivariable cox regression analyses predicting overall survival in patients with carcinoma of the bladder with variant histology within the SEER between 2004 and 2015.

		Univariable cox regression analyses		Multivariable cox regression analyses	
Histologic type	Overall mortality No. (%)	HR (95% CI)	*P*	HR (95% CI)	*P*
**Transitional cell carcinoma**					
No PLND	1232 (58.7)	1	<0.001	1	<0.001
PLND	6440 (49.2)	0.830 (0.781–0.882)		0.595 (0.557–0.634)	
**Squamous cell carcinoma**					
No PLND	68 (70.1)	1	0.010	1	0.002
PLND	355 (59.4)	0.710 (0.548–0.921)		0.646 (0.494–0.846)	
**Adenocarcinoma**					
No PLND	25 (58.1)	1	0.593	1	0.810
PLND	119 (55.3)	1.125 (0.730–1.733)		1.057 (0.671–1.667)	
**Small cell carcinoma**					
No PLND	9 (60.0)	1	0.325	1	0.136
PLND	94 (55.6)	0.709 (0.357–1.406)		0.576 (0.279–1.190)	
**Neuroendocrine carcinoma**					
No PLND	1 (20.0)	1	0.229	1	0.143
PLND	27 (50.9)	3.408 (0.461–25.177)		6.217 (0.540–71.560)	
**Signet ring cell carcinoma**					
No PLND	12 (85.7)	1	0.242	1	<0.001
PLND	60 (70.6)	0.690 (0.371–1.284)		0.233 (0.107–0.504)	
**Pseudosarcomatous carcinoma**					
No PLND	4 (44.4)	1	0.289	1	0.992
PLND	34 (75.6)	1.759 (0.619–4.997)		0.994 (0.307–3.219)	
**Other**					
No PLND	22 (61.1)	1	0.378	1	0.324
PLND	72 (60.0)	0.807 (0.500–1.301)		0.727 (0.386–1.369)	
**Overall**					
No PLND	1373 (59.3)		<0.001	1	
PLND	7201 (50.1)	0.833 (0.786–0.882)		0.601 (0.565–0.639)	<0.001

*95% CI, 95% confidence interval; HR, hazard ratio; PLND, pelvic lymph node dissection.*

## Discussion

Increasing data suggests that adding PLND to cystectomy provides considerable benefits. Specifically, the survival benefit of PLND versus non-PLND cohorts was emphasized in a systematic review (11). This review included twenty-three studies which suggested that removal of lymph nodes in bladder cancer surgery was beneficial and might result in better outcomes in terms of prolonging survival (11). However, most studies are based on analysis of urothelial (transitional cell) carcinoma of bladder. Most often, those individuals with non-urothelial bladder tumors have been excluded from clinical trials. This severely limits the application of PLND to non-urothelial bladder tumors.

Although previous evidence supports the use of PLND for bladder cancer cases receiving cystectomy, literature about the impact of PLND on OS stratified by histologic type is scarce (10). Thus, to further explore this area, we compared the OS between cases who underwent PLND or not stratified according to histologic type. To the best of our knowledge, this is the first large-scale study to evaluate the impact of histologic type on survival outcomes after cystectomy at a broader level. Our analyses confirmed a substantial OS benefit for cystectomy and PLND in patients with transitional cell carcinoma, squamous cell carcinoma, and signet ring cell carcinoma compared with patients receiving only cystectomy. However, we did not observe a survival advantage in other histologic types between the treatment groups.

Non-urothelial bladder cancers are a heterogeneous group that behave differently from urothelial bladder cancer. Lughezzani et al. reported that the incidence of lymph node invasion was significantly higher in adenocarcinoma when compared with urothelial carcinoma (26.5% vs. 21.7%; *P* = 0.04) (12). Abdollah et al. found that the rate of non-organ confined bladder cancer was higher in cases with squamous cell carcinoma than in their urothelial carcinoma counterparts (13). Both of these findings suggest that non-urothelial cancers are more advanced at diagnosis. In this large cohort of cases with bladder cancer, the incidences of lymph node invasion at the time of cystectomy range from 17.8 % (pseudosarcomatous carcinoma) to 58.8% (signet ring cell carcinoma). Noteworthy, the histologic type was significantly associated with the incidences of lymph node invasion. Consequently, the benefits of lymphadenectomy in patients with variant histology might differ due to the varied rates of lymph node invasion.

With regard to transitional cell carcinoma, the results of the present study are consistent with mounting evidence supporting the use of PLND during cystectomy (5, 14–16). May et al. observed that removal of a higher lymph node count was associated with improved survival in cases undergoing cystectomy (16). Moreover, the estimated OS for cases with a lymph node density of <20% was higher than those with a lymph node density >20% (16). Accordingly, the use of PLND during cystectomy for bladder cancer patients with transitional cell carcinoma is justified.

It has been reported that squamous cell carcinoma appeared to be more aggressive than urothelial carcinoma of bladder (17). Moreover, compared to cases with conventional urothelial carcinoma, survival outcomes were significantly worse in cases with squamous cell carcinoma (18). In addition, Honma et al. found that a concomitant squamous cell carcinoma component in the specimen was an independent predictor of local recurrence in patients treated with cystectomy (19). Although these studies have reported an association between squamous cell carcinoma and survival outcomes after cystectomy, the impact of PLND on OS remains controversial. Our Cox regression analyses revealed improved OS for patients with squamous cell carcinoma who underwent both cystectomy and PLND.

Adenocarcinoma is the third most common bladder cancer, and are generally divided into three categories: primary vesical, urachal, and metastatic lesions (20). Lughezzani and colleagues found that adenocarcinoma patients receiving cystectomy had more advanced disease stages. Moreover, adenocarcinoma was associated with worse prognosis than urothelial carcinoma after adjustment for all covariates (12). Wright et al. observed that the 5-year survival rate was less than 50% for patients with adenocarcinoma of the bladder (21). Despite the poor prognosis, it remains unclear if PLND could improve survival in these patients. Noteworthy, cases with adenocarcinoma of bladder appeared not to benefit from PLND with regard to OS.

Small cell carcinoma of the bladder is a rare condition, accounting for roughly 1% of patients receiving cystectomy (22). It is an aggressive tumor with a propensity for early metastasis. Considering that small cell carcinoma is mainly a systemic disease, cystectomy for local therapy is not satisfying. We also found that PLND was not independent predictor of OS in cases with small cell carcinoma of the bladder. Thus, chemotherapy may be essential to improve survival outcomes (23). According to the available evidence, it appeared reasonable to treat these patients with chemotherapy followed by cystectomy (24).

Signet ring cell carcinoma of the bladder is also rare malignancy. Akamatsu et al. reported that the OS rate for signet ring cell carcinoma at 2 years was 43%, and all cases with stage IV disease were dead at 2 years (25). Lendorf et al. conducted an updated review on primary signet ring cell carcinoma of the bladder, clearly demonstrating the poor prognosis of this disease and the imperative need for multi-institutional clinical trials to improve survival for these patients (26). For cases who had signet ring cell carcinoma of the bladder, our multivariable Cox regression analyses showed significant OS benefit for cystectomy and PLND compared with patients undergoing cystectomy only. Thus, with cystectomy and PLND, it might be probable to improve OS in bladder cancer patients with signet ring cell carcinoma.

A particular strength of the present study is the relatively large number of cases with variant histology compared with small, single-institutional studies. Thus, we could compare different histologic types of bladder cancer instead of concentrating on a single, specific entity. Moreover, we were able to reduce possible selection bias in baseline characteristics with the use of multivariable Cox regression models.

Our study has several limitations. First, the evaluation of histologic type was based on coding and not on a central pathologic slide review. Heterogeneity might occur in pathologic reporting due to difference experience of pathologists. Second, we only focused on whether patients received PLND, because the SEER database does not provide details in terms of PLND template. Third, although many histologic types have been reported within the SEER database, we summarized into eight major categories for assessing the role of PLND during cystectomy. For example, papillary and micropapillary transitional cell carcinoma were classified as transitional cell carcinoma. Of note, PLND might be associated with survival differences among patients with different histologic subtypes. Fourth, the SEER database does not provide information regarding neoadjuvant and adjuvant chemotherapy (27, 28), which could potentially have influenced oncologic outcomes. Fifth, there are inherent limitations which are associated with the observational and retrospective study design, because unclear confounding factors and potential selection bias might limit the generalizability of the findings. Sixth, there is an important risk of bias inherent to database studies. Seventh, there is no sensitivity analysis to assess unmeasured confounders, given absence of key confounding variables from the database. Despite these limitations, we used data from SEER database and gained insights into the impact of PLND on bladder cancer patients with different histologic type.

In conclusion, variant histologic types were significantly associated with the presence of lymph node invasion in patients with bladder cancer. We compared the effect of PLND on OS according to histologic type. Multivariable Cox regression analyses revealed a significant OS benefit from PLND in patients who had transitional cell carcinoma, squamous cell carcinoma, and signet ring cell carcinoma. However, a survival benefit of PLND in bladder cancer patients with other histologic types was not demonstrated. The findings should be interpreted within the context of the retrospective study design. Considering the small number of bladder cancer patients with rare histologies, additional prospective studies are needed to better elucidate the role of PLND in bladder cancer patients with histological variants.

## Data Availability Statement

All datasets presented in this study are included in the article/Supplementary Material.

## Author Contributions

XZ and ZZ: conceptualization. LG, LZ, and JW: methodology. LG and LZ: writing. XZ: supervision. ZZ: funding acquisition. All authors contributed to the article and approved the submitted version.

## Conflict of Interest

The authors declare that the research was conducted in the absence of any commercial or financial relationships that could be construed as a potential conflict of interest.
